# Genome assembly and analysis of *Lactuca virosa*: implications for lettuce breeding

**DOI:** 10.1093/g3journal/jkad204

**Published:** 2023-09-23

**Authors:** Wei Xiong, Dirk-Jan M van Workum, Lidija Berke, Linda V Bakker, Elio Schijlen, Frank F M Becker, Henri van de Geest, Sander Peters, Richard Michelmore, Rob van Treuren, Marieke Jeuken, Sandra Smit, M Eric Schranz

**Affiliations:** Biosystematics Group, Wageningen University & Research, P.O. Box 16, Wageningen, 6700 AA, The Netherlands; Bioinformatics Group, Wageningen University & Research, P.O. Box 633, Wageningen, 6700 AP, The Netherlands; Biosystematics Group, Wageningen University & Research, P.O. Box 16, Wageningen, 6700 AA, The Netherlands; Bioscience, Wageningen University & Research, P.O. Box 16, Wageningen, 6700 AA, The Netherlands; Bioscience, Wageningen University & Research, P.O. Box 16, Wageningen, 6700 AA, The Netherlands; Biosystematics Group, Wageningen University & Research, P.O. Box 16, Wageningen, 6700 AA, The Netherlands; Laboratory of Genetics, Wageningen University & Research, P.O. Box 16, Wageningen, 6700 AA, The Netherlands; Bioscience, Wageningen University & Research, P.O. Box 16, Wageningen, 6700 AA, The Netherlands; Bioscience, Wageningen University & Research, P.O. Box 16, Wageningen, 6700 AA, The Netherlands; The Genome Center, Genome & Biomedical Sciences Facility, University of California, Davis, 451 East Health Sciences Drive, Davis, CA 95616-8816, USA; Centre for Genetic Resources, the Netherlands (CGN), Wageningen University & Research, P.O. Box 16, Wageningen, 6700 AA, The Netherlands; Plant Breeding Group, Wageningen University & Research, P.O. Box 386, Wageningen, 6700 AJ, The Netherlands; Bioinformatics Group, Wageningen University & Research, P.O. Box 633, Wageningen, 6700 AP, The Netherlands; Biosystematics Group, Wageningen University & Research, P.O. Box 16, Wageningen, 6700 AA, The Netherlands

**Keywords:** lettuce, genome assembly, comparative genomics, transposable elements (TEs), immune genes, Plant Genetics and Genomics

## Abstract

Lettuce (*Lactuca sativa* L.) is a leafy vegetable crop with ongoing breeding efforts related to quality, resilience, and innovative production systems. To breed resilient and resistant lettuce in the future, valuable genetic variation found in close relatives could be further exploited. *Lactuca virosa* (2*x* = 2*n* = 18), a wild relative assigned to the tertiary lettuce gene pool, has a much larger genome (3.7 Gbp) than *Lactuca sativa* (2.5 Gbp). It has been used in interspecific crosses and is a donor to modern crisphead lettuce cultivars. Here, we present a de novo reference assembly of *L. virosa* with high continuity and complete gene space. This assembly facilitated comparisons to the genome of *L. sativa* and to that of the wild species *L. saligna*, a representative of the secondary lettuce gene pool. To assess the diversity in gene content, we classified the genes of the 3 *Lactuca* species as core, accessory, and unique. In addition, we identified 3 interspecific chromosomal inversions compared to *L. sativa*, which each may cause recombination suppression and thus hamper future introgression breeding. Using 3-way comparisons in both reference-based and reference-free manners, we show that the proliferation of long-terminal repeat elements has driven the genome expansion of *L. virosa*. Further, we performed a genome-wide comparison of immune genes, nucleotide-binding leucine-rich repeat, and receptor-like kinases among *Lactuca* spp. and indicated the evolutionary patterns and mechanisms behind their expansions. These genome analyses greatly facilitate the understanding of genetic variation in *L. virosa*, which is beneficial for the breeding of improved lettuce varieties.

## Introduction

Lettuce (*Lactuca sativa* L.) is a crop with an economic value of ∼3 billion USD per year (Food and Agriculture Organization of the United Nations 2019). To develop better lettuce cultivars, breeders often search for novel genetic variations in lettuce wild relatives. *Lactuca virosa* (biennial) is a donor for resistance to different pests and pathogens and a representative species in the lettuce gene pool (Maisonneuve *et al.* 1991, 2018; Maisonneuve 2003; Parra *et al.* 2016). The exploitation of *L. virosa* for lettuce breeding has had both challenges and successes. For example, despite reproductive barriers for direct intercrossing with lettuce, breeders and scientists were able to execute interspecific hybridization bridged by *L. serriola* to introduce traits like robust root architecture and resistance to currant-lettuce aphid, downy mildew, and viruses (Thompson and Ryder 1961; Eenink *et al.* 1982; Maisonneuve *et al.* 1995). Such interspecific crosses are part of the breeding pedigrees of the well-known cultivars Vanguard and Salinas, representing modern crisphead lettuce cultivars (Mikel 2007, 2013). Novel introgressions of desired genes and traits from *L. virosa* into cultivated lettuce could be realized through an improved understanding of its genomic content.

A reference genome and derived molecular markers are essential for breeders to select traits accurately and trace introgressions in cultivated lettuce from *L. virosa*. For example, genome-wide association studies (GWAS) have been performed to identify SNP variants (Mikel 2013) that are associated with interesting traits in lettuce (Walley *et al.* 2017; Sthapit Kandel *et al.* 2020; Simko *et al.* 2022) using the assembled lettuce (*L. sativa*) reference genome (Reyes-Chin-Wo *et al.* 2017), which can be used to develop markers for lettuce breeding to accelerate selection in offspring (Simko 2013). In addition to GWAS, a reference genome of *L. virosa* will also facilitate genomic analyses for various biological questions. A whole-genome screening can search genetic determinants, for instance, that trigger a resistance response. Genome rearrangements can be detected between *L. virosa* and other *Lactuca* species via comparative genomics.

The creation of a reference genome for *L. virosa* is challenging, because, even though it is a diploid species (2*n* = 2*x* = 18), it has a considerably larger genome (3.7 Gbp) than *L. sativa* (2.5 Gbp) and *L. saligna* (2.3 Gbp) (Doležalová *et al.* 2002). This is likely due to transposable elements (TEs) (Wendel *et al.* 2016). To date, there is only a single available genome assembly of *L. virosa* (CGN04683) (Wei *et al.* 2021), which is a short-read based and highly fragmented assembly (3,694,810 scaffolds; N50 = 4,910 bp) with relatively high completeness (BUSCO (Benchmarking Universal Single-Copy Orthologue) = 92.7%). Long-read sequencing could significantly improve the accuracy and continuity of a *L. virosa* genome assembly.

Here, we present a near chromosome-level de novo assembly of *L. virosa* (CGN04683) using a combination of long-read and short-read sequencing plus Bionano and Dovetail scaffolding. We contextualize the *L. virosa* genome within the lettuce gene pool together with the *L. sativa* and *L. saligna* (Xiong *et al.* 2023) genomes. First, we show shared and specific homology groups across the 3 species. Based on homologs, we show interspecific collinearity with an emphasis on inversions in different chromosomes. Next, we demonstrate that the proliferation of long-terminal repeat (LTR) superfamilies underlies the genome expansion of *L. virosa*. Finally, we describe a well-classified inventory of the 2 important resistance-related gene types encoding nucleotide-binding (NB) leucine-rich repeat (NLR) receptors and receptor-like kinases (RLK).

## Materials and methods

### DNA and RNA sequencing

*L. virosa* accession CGN04683, is also known as IVT280 and is resistant to *Nasonovia ribisnigri* (currant-lettuce aphid) (Eenink *et al.* 1982). Single seed descent of accession “IVT280” (seeds obtained from a breeding company) was grown for whole-genome sequencing. The seeds were stratified at 4°C for 3 days to improve germination. Subsequently, seedlings were grown in a growth chamber at 18–21°C and a relative humidity of 75–78%. After 8 weeks, plants were transplanted to larger pots containing potting soil and grown under greenhouse conditions. Tissue sampling was performed when plants were close to bolting, and DNA was extracted using the same protocol described in Xiong *et al*. (2023). DNA material was used to prepare libraries with the SMRTbell Template Prep Kit 1.0 and SMRTbell Damage Repair Kit. For library construction, we used the Procedure & Checklist –Preparing >30 kb SMRTbell Libraries Using the Megaruptor Shearing and BluePippin Size-Selection System. Then, we produced a 20-fold coverage of long-read data generated by PacBio Sequel technology using 20 SMRT cells. For the Illumina data, the Illumina TruSeq DNA Sample Preparation kit was used. Then, mechanical DNA shearing using Covaris E210, Illumina TruSeq DNA Sample Preparation Guide. Flowcell cluster generation was done using an Illumina cBot device, sequencing was done using an Illumina HiSeq2000 platform. We used an insertion size of 500 bp and a read length of 125 bp to obtain a 69-fold coverage of paired-end (PE) reads. An optical mapping library of 130 × coverage was produced by Bionano mapping for hybrid scaffolding. For Bionano, DLE-1 (Direct Label Enzyme) labeling enzyme was used at a density of 17.54/100 kbp. The Bionano Genomics Direct Label and Stain (DLS) Kit was used and 30206-Bionano-Prep-Direct-Label-and-Stain-DLS-Protocol_rev D was used for library construction. A Hi-C library produced by Dovetail Genomics provided 10,492 × physical coverage of the genome (10 kbp–10 Mbp pairs) for in vitro proximity ligation (Supplementary Table 1). Finally, 10X sequencing was performed as well with the DNA material (150 bp read length). For this, we used the Chromium Genome Library, Gel Bead & Multiplex kit. Libraries were subsequently constructed using the Chromium Genome Reagent Kits User Guide, using a 10X Genomics Chromium controller. As additional evidence for gene prediction, RNA was isolated from pooled samples of leaf, root, and flower tissues (pooled from different floral stages) using a Direct Zol RNA Miniprep Plus kit (Zymo Research) followed by treatment with DNAse. RNA was purified by ethanol precipitation. The concentration and purity of RNA samples were measured with a Nanodrop 2000c spectrophotometer and a Qubit 4.0 fluorometer using an RNA Broad Range assay (Thermo Fisher Scientific). PE sequencing (2 × 125 bp) was performed on an Illumina HiSeq2500 platform (Supplementary Table 1). All library preparation, construction, and sequencing were performed in-house at the genomics facility of Wageningen University and Research Business Unit Bioscience.

### Genome assembly and annotation process

#### Genome size estimation

After trimming, PE Illumina reads of *L. virosa* were used for genome size estimation (∼1,590 million reads; ∼183 Gb). Jellyfish v2.3.0 was used with a k-mer size of 21 to count k-mer frequencies (maximum 1 million count) (Marçais and Kingsford 2011). The Jellyfish output was used by GenomeScope (v1.0) to estimate haploid genome length, percentage of repetitive DNA, and heterozygosity of the *L. virosa* genome (Ranallo-Benavidez *et al.* 2020).

#### Genome assembly

PacBio reads were assembled using Canu (v1.3) and then polished by Pilon (v1.20) using Illumina data (Walker *et al.* 2014; Koren *et al.* 2017). Then, we performed hybrid scaffolding for the assembly using the Bionano optical mapping data with the Bionano solve software. Mis-joins in assembled contigs were corrected using the HiRise pipeline with the Hi-C data (Putnam *et al.* 2016). Since the resulting assembly of Hi-C scaffolding was only 75.2% BUSCO complete, the publicly available—but highly fragmented—assembly for *L. virosa* (Wei *et al.* 2021) was used to augment the completeness of our assembly. The newly generated PE Illumina reads were trimmed before use with Trimmomatic v0.39 ILLUMINACLIP:TruSeq3-PE.fa:2:30:10 LEADING:3 TRAILING:3 SLIDINGWINDOW:4:15 MINLEN:36 (Bolger *et al.* 2014), and the barcodes of the 10X mate pairs were stripped with Longranger v2.2.2 basic. Before combining the assemblies, we first polished our assembly for a second time with the PE Illumina reads and the 10X mate pair reads (treated as single-end reads) using Pilon v1.24 –changes –diploid –fix all (Walker *et al.* 2014). Mapping of sequencing reads for combining these 2 assemblies was performed with bwa-mem2 v2.2.1 (Vasimuddin *et al.* 2019). Next, we combined our assembly with all sequences >1 kb in the Wei *et al*. (2021) assembly by concatenating the 2 genome assemblies. We then aligned all PE Illumina and 10X data to the combined genome. The coverage of this data was used to get the best haplotype representation of the complete genome with purge_haplotigs v1.1.1 (cutoffs were 10, 85, 180) (Roach *et al.* 2018). Since the number of sequences in the resulting assembly increased from 29 to 54,814, we applied several filtering steps to reduce the number of small, uninformative sequences. We filtered out possible mitochondrial and plastid sequences by blasting (BlastN) all sequences to the mitochondrial and plastid NCBI databases (*dd.* 2021 August 3). We filtered out non-*Viridiplantae* sequences as identified by a Blastn search against the NCBI database. Then, we polished the newly added sequences using the same method we used to polish our original genome assembly before (with Pilon v1.24). Based on coverage of PE Illumina and 10X data, we used purge_haplotigs to check whether any duplications were introduced, but since this was not the case, we did not apply purge_haplotigs a second time. For scaffolding the newly added sequences, we mapped the original PacBio data to the genome with minimap2 v2.21-cxmap-pb (Li 2018). Scaffolding was done with LRScaf v1.12-misl 3-t mm (Qin *et al.* 2019). To keep only potential gene coding sequences, we mapped the RNA-seq data with STAR v2.7.7a (Dobin *et al.* 2013) and removed all sequences lacking a single alignment. Finally, we also removed all sequences smaller than 5 kb.

#### Assessment of genome completeness

Genome and proteome (annotation) completeness were assessed using BUSCO v5.2.0 with the “eudicots_odb10” dataset (Manni *et al.* 2021). K-mer completeness was assessed with KAT v2.4.1 with a k-mer value of 31 (Mapleson *et al.* 2016).

#### Repeat annotation

To annotate the repetitive elements in the *L. virosa* genome, a custom library was created by combining different sources: a de novo library of TEs created by RepeatModeler (v2.0.1) with -LTRStruct parameter, a de novo library of miniature inverted-repeat transposable elements (MITEs) searched by MITE-hunter, and a specific database for the genus *Lactuca* extracted from a combined database of Dfam (20170127) and Repbase (20170127) (Han and Wessler 2010; Bao *et al.* 2015; Hubley *et al.* 2016; Flynn *et al.* 2020). Then, RepeatMasker (v4.0.7) was used to soft mask the *L. virosa* genome assembly (Smit *et al*. 2019). The same pipeline was also applied to create a TE library and mask the genome assembly of *L. saligna* version 4 (PRJEB35809) and *L. sativa* version 7 (GCF_002870075.2), which were used in reference-based repeatome comparison. The 3 generated TE libraries were used for a reference-free approach to TE classification (see Individual and comparative clustering analysis of repetitive elements below). The RepeatMasker outputs were further processed to summarize the different categories of repeat elements. Moreover, the LTR elements were extracted from the cross_match output of RepeatMasker and compared to the genome to determine their relationship to the genetic regions using bedmap in BEDOPS toolkit (v2.4.40) (Neph *et al.* 2012).

#### Gene prediction

Protein-encoding genes in the nuclear assembly were annotated using MAKER2, which combines de novo gene prediction and homology prediction (Holt and Yandell 2011). rRNA reads were filtered out from the RNA-seq data by SortMeRNA version 4.3.4 (Kopylova *et al.* 2012) using all databases to remove noncoding rRNA. Subsequently, HISAT2 (v2.2.1) was applied to map the remaining RNA-seq reads to the final genome assembly, which includes nuclear sequences, the mitochondrion assembly of CGN013357 (MZ159960.1), and plastid assembly of TKI-404/CGN04683 (CNP0000335 on Chinese National GeneBank (CNGB)) (Kim *et al.* 2015; Fertet *et al.* 2021; Wei *et al.* 2021). The alignment to the nuclear sequences was used as input to BRAKER (version 2) and Stringtie (v2.1.6) to conduct de novo gene prediction and transcriptome assembly, respectively, both with default settings (Pertea *et al.* 2015; Hoff *et al.* 2016). The protein alignment was done by BLAST in MAKER2 during the integration with protein databases of *A. thaliana* (Araport11), *L. sativa*, *Helianthus annuus* (HA412.v1), and Uniprot (SwissProt set only: release-2019_10). The predicted transcripts were then filtered using the following criteria: eAED >0.9 (computed by MAKER2), protein length <50, identical isoforms, and missing start and stop codon.

#### Functional annotation

Potential biological function of proteins was inferred using 3 criteria: (1) best-hit matches in SwissProt, TrEMBL using DIAMOND version 2.0.14 at E-value cutoff of 1e-5 (Buchfink *et al.* 2015); (2) protein domains/structure identified by InterProscan 5.53–87.0 against the Pfam, Coils, Gene3D, PANTHER, SUPERFAMILY, ModiDBLite, and TIGRFAM databases (Zdobnov and Apweiler 2001; El-Gebali *et al.* 2019); and (3) orthology searches for pathway information were conducted by Kofamscan (Aramaki *et al.* 2020) using a customized HMM database of KEGG orthologs (Kanehisa 2000) with an E-value cutoff of 1e-5.

### Homology analysis

#### Gene space analysis

To enable a comparison between *L. virosa*, *L. saligna*, and *L. sativa*, we used PanTools v3.4.0 (Jonkheer *et al.* 2022) to calculate homologous relationships in a predicted panproteome of these 3 species. We used the longest isoform for each gene. Based on an optimal distribution of BUSCO genes, we decided to use “pantools group -rn 2” for homology grouping. Subsequent gene classification of the homology groups was also done with PanTools. The number of shared groups was visualized with ComplexUpset (Krassowski 2020). Functional enrichment analyses were performed and visualized for the unique sets of genes with ClusterProfiler v3.18.1 (Yu *et al.* 2012).

#### Synteny detection

MCScanX (Wang *et al.* 2012) was utilized to detect syntenic blocks (default settings) among the 3 *Lactuca* species using the calculated homology groups from PanTools v3.4.0. The interspecific collinearities were visualized using SynVisio (Bandi and Gutwin 2020). MCScanX was run a second time to detect the tandem arrayed genes using DIAMOND (version 2.0.14) on proteomes for each species.

### Individual and comparative clustering analysis of repetitive elements

RepeatExplorer2 on a Galaxy server was used (https://repeatexplorer-elixir.cerit-sc.cz/) to conduct individual and comparative clustering of Illumina PE reads (all trimmed to a length of 120 bp) for 3 *Lactuca* species (*L. sativa*, *L. saligna*, and *L. virosa*) (Novák *et al.* 2020). Resequencing data of these 3 *Lactuca* species were retrieved from the European Nucleotide Archive (ENA) database (PRJEB36060). Trimmed FASTQ reads were converted to FASTA format and interlaced before the clustering analysis. In addition, a 4-letter prefix identity code was added to each sample dataset (i.e. Lsat for *L. sativa*, Lsal for *L. saligna*, and Lvir for *L. virosa*). After a preliminary round, each set of reads was randomly subsampled with the same proportion to maximize the repeat detection and annotation accuracy. For individual analysis, reads representing 20% of the genome size were separately clustered for each *Lactuca* species (i.e. genome proportion = 0.2X, *L. sativa* = 4,166,668 reads, *L. saligna* = 3,833,334 reads, and *L. virosa* = 6,166,668 reads). For comparative analysis, a mixed dataset of reads equal to 0.07 × depth for all species were clustered at once (i.e. genome proportion = 0.07 ×, *L. sativa* = 1,307,006 reads, *L. saligna* = 1,420,966 reads, and *L. virosa* = 2,103,018 reads). For both analyses, the reads were clustered based on the default settings (90% similarity, 55% coverage), and clusters containing more than 0.01% reads were classified at a supercluster level.

After clustering, repeat reads were annotated based on a similarity search to REXdb (protein domain in retrotransposons, Viridiplantae version 3) (Neumann *et al.* 2019) using BLAST on the Galaxy server. Additionally, the custom libraries previously created by reference-based searches were utilized as an additional custom library to further annotate the repeat clusters (see previous section: Repeat annotation). After annotation, clusters from plastid and mitochondrial origins were identified and excluded for downstream analysis. Next, we quantified different TE categories based on clusters and their connections to superclusters. To characterize the interspecific difference, the clusters resulting from the comparative analysis were sorted via hierarchical clustering (ward.D2) using transformed read number [log_2_(count + 1)] in each cluster for every species.

### Analysis of immune gene repertoire

NLRs were searched for in the predicted proteomes of *L. virosa* and *L. sativa,* and retrieved from the *L. saligna* genome (Xiong *et al.* 2023). HMMER v3.3.2 (Finn *et al.* 2011) was used to search Hidden Markov Models (HMMs) profiles obtained from Pfam or the UC Davis database for structural domains of NLR proteins (E-value cutoff = 1e-10): PF00931.23 and NBS_712.hmm (https://niblrrs.ucdavis.edu/At_RGenes/HMM_Model/HMM_Model_NBS_Ath.html) for the NB domain; PF01582.20 and PF13676.6 for TIR (TOLL/interleukin-1 receptor); PF05659.11 and PF18052.1 for CC (coiled-coil); and 8 HMMs for the LRR (leucine-rich repeat) domain (PF00560.33, PF07723.13, PF07725.13, PF12799.7, PF13306.6, PF13516.6, PF13855.6, PF14580.6). Furthermore, NB and LRR domains identified by InterProScan (see Functional annotation), and CC motifs predicted by Paircoil2 (*P* scores <0.025) were combined with the HMMER output (Zdobnov and Apweiler 2001; McDonnell *et al.* 2006). The identified NLRs were classified as TNL [Toll-interleukin-1 receptor-like NB site Leucine-rich repeat (NBS-LRR)] or CNL [Coiled Coil (CC), Resistance to powdery mildew8 (RPW8), or potato R protein domain (Rx_N) NBS-LRR] based on the presence of either the TIR or CC domain, respectively. To further solve the unclassified NLRs (TNL or CNL), a phylogenetic tree for amino-acid (aa) sequences with NB domains was constructed. First, aa sequences were aligned using HmmerAlign (Finn *et al.* 2011). The alignment was then trimmed by trimAl using -automated1 mode and retained 727 residues for phylogenetic construction (Capella-Gutiérrez *et al.* 2009). A maximum-likelihood (ML) tree was inferred by IQTREE version 1.6.12 (-m PMB + F + R10) with 1,000 ultrafasta bootstrap (UFBoot) replicates (Nguyen *et al.* 2015). The phylogenetic tree was visualized and annotated using iTOL v6 (Letunic and Bork 2021).

An Inventory of RLKs was also performed for *L. virosa* and *L. sativa*. First HMMER (v3.3.2) was used to search the Pkinase domain (PF00069; E-value cutoff = 1e-10). Then, proteins containing Pkinase were examined for the existence of extracellular domains using HMMER (E-value cutoff = 1e-3) and transmembrane regions using TMHMM (v2.0) and SCAMPI (v2) (Krogh *et al.* 2001; Peters *et al.* 2016).

## Results and discussion

### Genome assembly and annotation

We created a complete and structurally informative genome assembly for *L. virosa* with a total length of 3.45 Gbp (Table 1). Based on a k-mer analysis of Illumina data, we estimated the genome size to be 3.3 Gbp with 73% repeat content and 0.169% heterozygosity rate (Supplementary Fig. 1). This predicted size was lower than the previously measured C-value (3.7 Gbp) (Doležalová *et al.* 2002), which might be caused by the large repeat content of *L. virosa* (Ranallo-Benavidez *et al.* 2020). The long-read assembly was based on PacBio and Illumina data and scaffolded using Bionano and Hi-C data. The longest 12 scaffolds out of the 29 scaffolds comprised 99.8% of the total length (3.3 Gbp) of this first assembly, yet not all chromosomes were reconstructed in full. Therefore, we completed the assembly through additional polishing and leveraging the fragmented, short-read-based genome assembly of the same *L. virosa* accession (Wei *et al.* 2021) which we combined in a nonredundant way (Supplementary Data 1 and Supplementary Figs. 2b and 2d). The final combined assembly consisted of 5,855 contigs spanning a total of 3.45 Gbp with an N90 score of 116,478,781 and an L90 score of 10 (Supplementary Fig. 2c and Table 2). The BUSCO completeness score was 96.2% (the duplication score was 4.5%; Supplementary Table 3 and Fig. 2d).

**Table 1.** Summary of assemblies for *Lactuca* spp. in this paper.

Characteristic	*L. sativa*	*L. saligna*	*L. virosa*
Accession ID	GCF_002870075.2	PRJEB35809	PRJEB50301
Source	RefSeq (NCBI)	ENA	This study
Assembly size (Gb)	2.39	2.17	3.45
# seq	8,325	10	5,855
N50 scaffold	257.9 Mb	238.6 Mb	316.9 Mb
L50 scaffold	4	4	5
Genome complete BUSCO	97.8% (2,273)	92.4% (2,147)	96.2% (2,236)
# protein-coding genes	36,136	42,908	39,887
# transcripts	46,867	45,476	42,791
Proteome complete BUSCO	98.5% (2,291)	88.8% (2,065)	90.2% (2,096)

Based on both expression and orthology evidence, 39,887 protein-coding genes with a total of 42,791 transcripts were annotated. We mapped RNA-seq data from root, leaf, and flower tissue to the genome assembly to support de novo gene prediction. Next, we aligned protein sequences of model plant species to the genome and used MAKER for merging all gene predictions. We filtered the predicted genes to only retain annotations that were in accordance with the provided evidence. The BUSCO score on the resulting proteome was 90.2%, indicating a high level of completeness. Furthermore, we were able to predict functional domains in 93% (37,106) of the genes for various databases (Supplementary Table 4 and Data 2a and 2b). This structural and functional annotation is vital for the biological interpretation of *L. virosa* data.

### Homology grouping of 3 representative *Lactuca* spp.

**Fig. 1.**
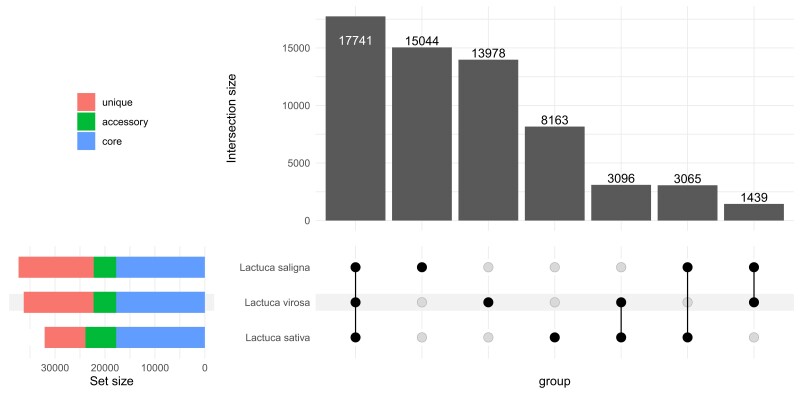
Overview of homology grouping for *L. sativa*, *L. saligna*, and *L. virosa* in an upset plot. The numbers on bars are groups of homologous genes. In total, there are 62,526 homology groups.

Even though the genome size of *L. virosa* is substantially larger than *L. sativa* and *L. saligna*, the number of genes annotated across species was similar (Table 1). A comparison of *L. virosa* with *L. saligna* and *L. sativa* showed that about half of the homology groups are shared across *Lactuca* (Fig. 1; Supplementary Data 2c). These 17,741 homology groups in *Lactuca* contained 19,270 *L. virosa* genes, meaning that about half of the *L. virosa* genes are part of the core *Lactuca* genome. This is comparable to what was found in other interspecies comparisons. For example, in rice ∼62% of core genes were reported between 2 species (Zhao *et al.* 2018), and in *Raphanus*, ∼50% of core genes were reported among 11 accessions belonging to 2 species (Zhang *et al.* 2021). Both *L. virosa* and *L. saligna* share fewer homologous genes with each other than with *L. sativa*. This stresses the importance of wild species in breeding as they contain a large pool of novel genes. The large, unique genomes of both *L. virosa* and *L. saligna* indicate that these wild species are rich sources of genetic diversity that thus far has been unexploited for lettuce breeding. We performed a functional annotation for the proteomes of the 3 species with InterProScan to perform functional enrichment for the unique content of *L. virosa* (15,048 genes; Supplementary Data 2d). The InterProScan domain enrichment found disease resistance proteins to be among the set of significantly enriched domains (Supplementary Fig. 3). Therefore, the genome of *L. virosa* is a resource for potential novel genes needed for resilience breeding in lettuce.

Furthermore, it will be relevant to sequence and produce high-quality assemblies of other wild relatives of lettuce, such as *L. georgica*, *L. serriola*, and *L. aculeata,* to obtain an overview of the entire *Lactuca* gene space (Wei *et al.* 2021; Guo *et al.* 2023). Using high-quality genetic resources will enable the construction of a comprehensive pangenome that covers the variation in the genus *Lactuca*.

### Synteny detection between 3 *Lactuca* spp. via comparative genomics

**Fig. 2.**
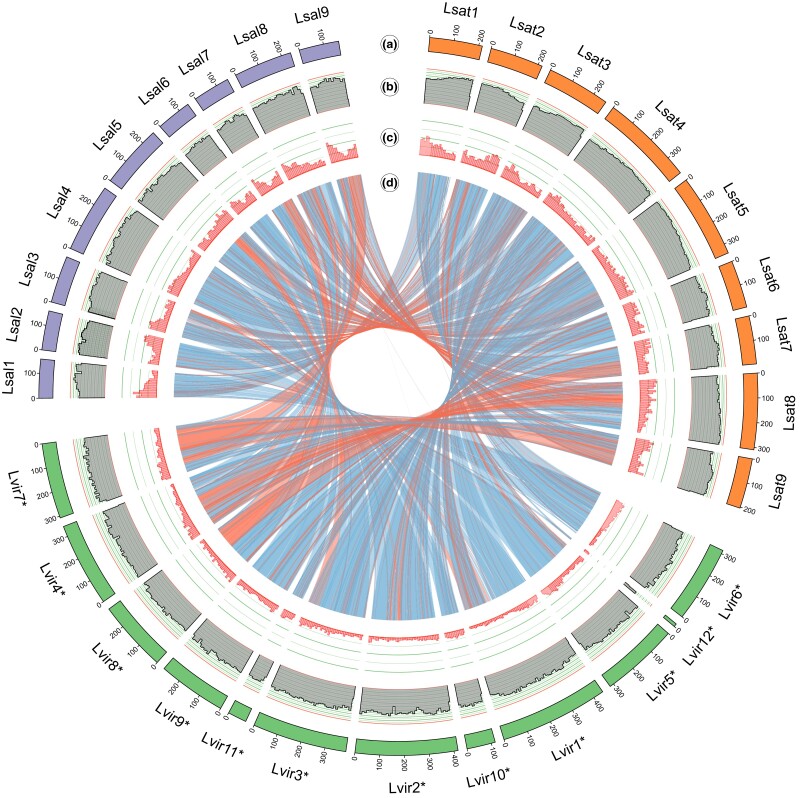
Circos plot of *L. virosa* genome compared with the *L. sativa* and *L. saligna* genomes. a) For each of the 3 genomes (Lsat: *L. sativa*; Lvir: *L. virosa*; Lsal: *L. saligna*), only sequences larger than 1 Mbp are shown. For *L. sativa* and *L. saligna*, the number of sequences correspond to their chromosomes. Since *L. virosa* is near-chromosome level, its sequence numbers were indicated with an asterisk (*). The sequences were sorted based on their collinearity to other *Lactuca* species, and some of the sequences for *L. virosa* are inverted to match the orientation in previously published genomes (d); the sequence coordinates (in Mbp) show this. b) The repeat density for each sequence is calculated per 10 Mbp and shown here as a fraction. Since the genome assembly for *L. virosa* has more N bases, repeats are more difficult to find than in the other 2 genomes. The scale ranges between 0 and 1. c) The gene density for each sequence is calculated per 10 Mbp and shown here as a fraction. As the 3 genomes contain approximately the same number of genes but their genome sizes differ, *L. virosa* has a lower overall gene density. The scale goes from 0 to 0.2. d) Synteny between the 3 genomes. Inversions are shown in red as opposed to noninverted syntenic blocks, which are shown in blue.

**Fig. 3.**
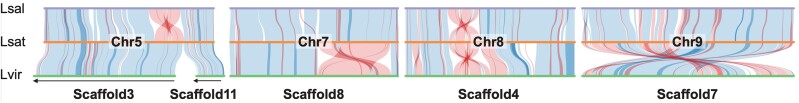
Synteny discloses species-specific inversions across 3 *Lactuca* species. Through genomic comparison, major interspecific inversions (red) were identified among the reference genomes of 3 *Lactuca* species. Here, the synteny in 4 sets of scaffolds/chromosomes reveals species-specific inversions: *L. saligna* (Lsal: purple), *L. sativa* (Lsat: orange), and *L. virosa* (Lvir: green). The chromosome numbers are labeled in the middle. Black arrows at the bottom indicate reversed scaffolds in the *L. virosa* assembly. Supported by Supplementary Table 5.

By synteny detection of homologous pairs, we identified major chromosomal inversions between the 3 *Lactuca* genomes. Overall, there was whole-genome collinearity (synteny) among *Lactuca* species (Fig. 2). Based on the collinearity, we determined the major 12 scaffolds that comprised 96% (3.30 Gbp) of the total genome assembly (Supplementary Table 5). Compared to the *L. sativa* genome, 3 species-specific inversions were identified on different chromosomes (Fig. 3). Two of the 3 inversions that were previously described between *L. saligna* and *L. sativa* were validated and further characterized: one is specific to *L. saligna* on Chr5 and one is specific to *L. sativa* on Chr8 (Xiong *et al.* 2023). Furthermore, synteny also revealed a large inversion specific to *L. virosa* on tentative Chr7 (Scaffold8) (Fig. 3; Supplementary Table 5). These inversions could hamper genetic mapping of interesting traits and further introgression. The syntenic patterns between *L. virosa* Chr9 (scaffold7) and the other 2 species showed complicated inverted and translocated regions, which might be due to a reversed joining indicated by the mapping of Hi-C data (Supplementary Fig. 4).

### Comparative repeatomics between 3 Lactuca spp. via reference-based and reference-free approaches

In the 3 reference assemblies, we annotated repeat elements and classified them into TEs and other repeats (Supplementary Data 3a). The genomes of all 3 *Lactuca* species contained a major proportion of TEs, in agreement with previous studies (Supplementary Table 6) (Simko *et al.* 2022). Unsurprisingly, the TE content of the *L. virosa* (60%) assembly is proportionally lower than that of both *L. sativa* (74%) and *L. saligna* (77%), which is likely caused by an incomplete search due to the high N content in the *L. virosa* assembly. After excluding the N content of each genome, the percentage of TEs for all *Lactuca* genomes exceeded 80% (Supplementary Fig. 5; Supplementary Table 6). Moreover, almost all identified LTRs (99%) were located in the intergenic regions (Supplementary Fig. 6). To conclude, this reference-based repeat annotation showed that TEs are the most abundant components of *Lactuca* spp. genomes. However, genome incompleteness and N content of the reference genome assemblies hamper a precise estimation of TEs.

**Fig. 4.**
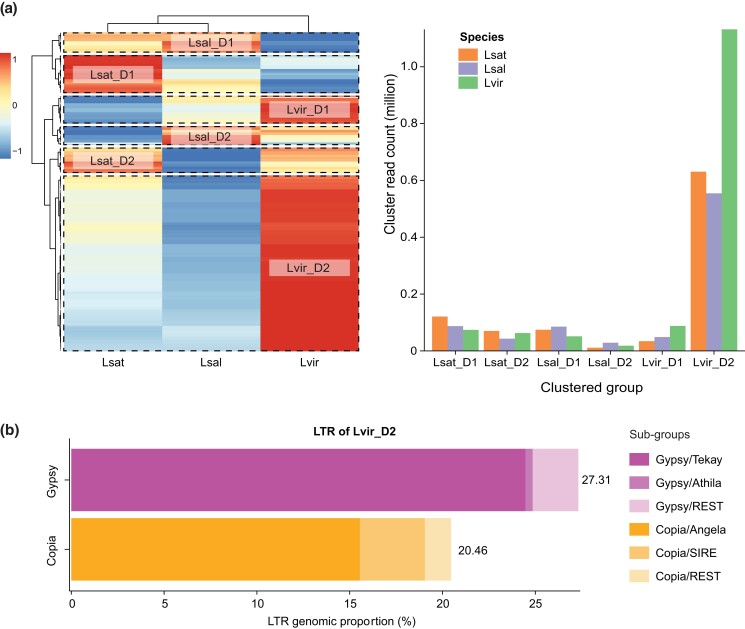
Proliferation of long-terminal repeats (LTR) drives the expansion of the *L. virosa* genome. Read clusters assembled by RepeatExplorer2 using a mix of resequencing data (coverage = 0.07×) from 3 *Lactuca* species references to detect the major difference of repeat elements. a) Heatmap shows the scaled read-count of individual cluster (row) for each species (column). Clusters and species were sorted by hierarchical clustering. Six groups (squared by dash lines) were dominated by reads either from *L. sativa* (Lsat), *L. saligna* (Lsal), or *L. virosa* (Lvir) and suffixed with a D (dominant). Bar plot shows the size (y-axis) of 6 clustered groups (x-axis) for each species: *L. sativa* (orange), *L. saligna* (purple), and *L. virosa* (green). b) A stacked bar chart shows the composition of subgroups for the 2 major LTR superfamilies: Gypsy (gradient purple) and Copia (gradient yellow). Supported by Supplementary Table 9.

In addition to reference-based repeat annotation, we also classified repeat components and estimated their composition for 3 *Lactuca* spp. using a reference-free approach. First, the same depth of reads (0.2×) were sampled and clustered for each species for repeat classification (Supplementary Tables 7 and 8). *L. virosa* had the highest percentage of repeated reads assembled as clusters (82%). The genomic proportion of repeated sequences annotated as TEs was more than 60% for all species, with *L. virosa* having the highest amount of LTRs (68.34%). Another comparative analysis (read depth = 0.07×) indicated that *L. virosa* carries a higher percentage of repeats compared to the other 2 species (Supplementary Data 3b and Table 8). For example, cluster 10 was annotated as LTR/Gypsy and mainly composed of *L. virosa* reads (Supplementary Fig. 7). Moreover, in-depth cluster analysis showed that LTR proliferation in *L. virosa* drove its genome expansion (Supplementary Data 3c and 3d). The heatmap of hierarchical clustering shows 6 groups that were either dominated (D) by one of the 3 *Lactuca* species: *L. sativa* (Lsat), *L. saligna* (Lsal), or *L. virosa* (Lvir) (Fig. 4a: left). The bar plot in Fig. 4a (right) further decomposes the read sources for each group. The Lvir_D2 group is the largest and dominated by *L. virosa* reads. This group mainly consisted of LTR subfamilies Gypsy (27.31%) and Copia (20.46%) (Supplementary Table 9). Additionally, the subgroups Tekay and Angela were the primary elements for the Gypsy and Copia clusters within the Lvir_D2 group (Fig. 4b; Supplementary Table 9).

*L. virosa* is estimated to have a significantly larger genome (3.7 Gbp) than *L. sativa* (2.5 Gbp) and *L. saligna* (2.3 Gbp) (Doležalová *et al.* 2002). TEs have been shown to drive plant genome expansion (Wendel *et al.* 2016); for example, within the genus of rice (Ma and Bennetzen 2004; Piegu *et al.* 2006; Ammiraju *et al.* 2007). Based on our combined findings, we conclude that the subgroups of transposon LTR, Tekay in Gypsy, and Angela in Copia drove the genome expansion of *L. virosa*.

### Comparison of NLR and RLK genes between 3 *Lactuca* spp.

Besides the difference within TEs, there is also sizable variation in the number of genes as shown by the homology grouping (accessory/unique genes) among these 3 *Lactuca* species (Supplementary Fig. 3), which might convey resilience to important traits like resistance against various pathogens or pests. In our previous study, an extensive search of resistance genes was performed for lettuce and its wild relative *L. saligna* (Xiong *et al.* 2023). Using the new *L. virosa* assembly, we identified and classified immunity-related genes encoding NLR and RLK proteins for *L. virosa* and compared them to *L. sativa* and *L*. *saligna*.

**Fig. 5.**
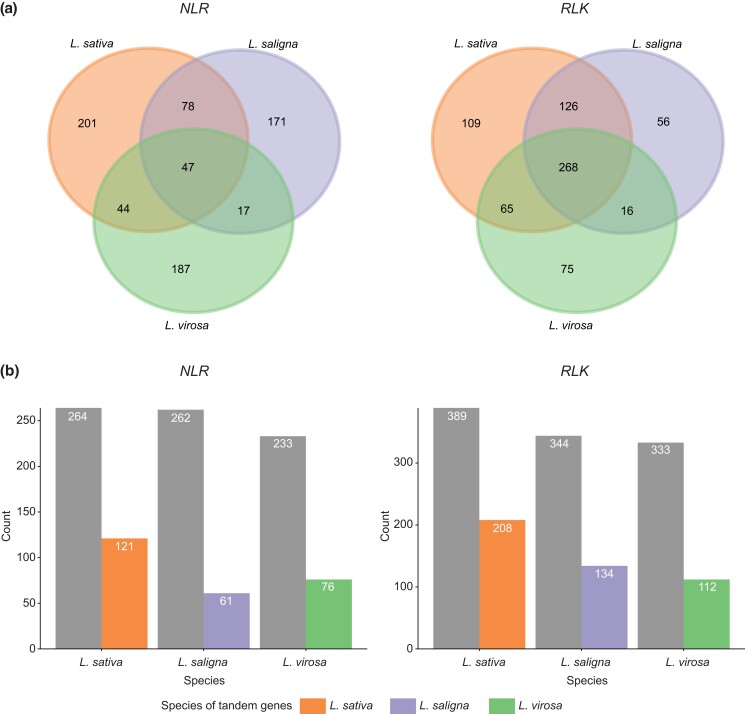
Homology and tandem duplication relationships of immune genes across *Lactuca* species. a) Venn diagrams of homology groups for NB leucine-rich repeats (*NLRs*) and *RLK*s in *Lactuca* spp. Homology grouping was done by PanTools. b) Bar plots show the count of tandem (colors) and nontandem (gray) *NLRs* and *RLKs* in 3 *Lactuca* species. Tandem-arrayed genes were identified by MCScanX. Supported by Supplementary Data 5.

The *L. sativa* genome was found to have the highest number of NLRs (385), followed by *L. saligna* (323), and *L. virosa* (309) (Table 2; Supplementary Table 10). In association with the homology grouping, a Venn diagram showed that the NLRs identified in 3 *Lactuca* spp. are highly diverged, where more than 50% of NLRs in each species belong to specific homology groups (Fig. 5a: left; Supplementary Data 5a). This observation is in line with our enrichment study of homologs specific to *L. virosa*, where InterProScan domains were significantly enriched with terms related to NLR proteins (Supplementary Fig. 3). Furthermore, NLR proteins were classified into TNL and CNL types based on the N-terminal domain (TIR or CC domain, respectively) and curated by the phylogeny of a NB domain alignment (Supplementary Fig. 8; Supplementary Data 4a and 4b). The difference between *L. sativa*, *L. saligna*, and *L. virosa* was mainly contributed to *TNL* genes (227 vs 184 and 180), and the difference between *L. saligna* and *L. virosa* can be explained by the *CNL* type (139 vs 162). Due to the unequal completeness of the proteomes, we applied the ratio of complete BUSCOs for proteomes as a benchmark to anticipate whether *NLR genes* expand or contract between the 3 *Lactuca* spp.: *L. sativa* (2,291), *L. saligna* (2,065), and *L. virosa* (2,096). The ratio of BUSCOs (1.10 : 1.00 : 1.02) reflects the NLR ratio across species (1.25 : 1.05 : 1.00), where *L. sativa* showed a slight inflation. For different NLR types, the number of CNLs was similar in the examined species *L. sativa*, *L. saligna*, and *L. virosa* (1.14 : 1.00 : 1.06); however, the ratio of TNL numbers highly deviated from the BUSCO ratio (1.41 : 1.14 : 1.00; Supplementary Table 10). Such comparison suggests an expansion of *NLR*s in *L. sativa*, which is possibly caused by tandem duplication events as in most studied angiosperms (Wu *et al.* 2021). This hypothesis is supported by a whole-genome search of tandem duplicates (TDs) clusters between 3 *Lactuca* spp. genomes (Supplementary Data 5b). The number of TDs encoding NLRs in *L. sativa* (121) was approximately 2-times larger than that in *L. saligna* (61) and *L. virosa* (76), which principally explains the number difference among the 3 species (Fig. 5b: left). In addition to tandem duplication, transposon activities (e.g. LTRs) could also greatly elevate the number of *NLR*s by retroduplication as reported in the chili genome (Kim *et al.* 2017). The retroduplicated *NLR*s could partially explain the lineage-specific homologs among *Lactuca* species (Fig. 5a: left).

**Table 2.** Identification and classification of candidate immunity-related genes for Lactuca spp.

Immune genes		Species		
Family	Classification	*L. sativa*	*L. saligna*	*L. virosa*
*NLR*	CNL*^a^*	158	139	148
	TNL	227	184	161
	Total	385	323	309
*RLK^b^*	Rcc1-RK	5	5	2
	WAK	61	48	36
	G-LecRK	132	79	70
	L-LecRK	31	29	21
	C-LecRK	1	1	1
	CRK	41	35	38
	Malectin-RK	55	55	32
	LysM-RK	12	12	11
	LRR-RK	258	213	233
	PERK	1	1	1
	Total	597	478	445

RPW8 and Rx_N type of CNL included in this study.

RLK classification based on the extracellular domain (Supplementary Tables 11 and 12).

We next identified RLK proteins by searching for the extracellular, transmembrane, and intracellular domains. Then, resulting RLKs were classified into 9 types based on their extracellular and kinase domains (Supplementary Tables 11 and 12 and Data 4c and 4d). Like NLRs, we found more genes encoding RLK proteins in the *L. sativa* (597) genome assembly than in *L. saligna* (478) or *L. virosa* (445; Table 2). Sequence similarity shows that *RLK*s were much more conserved in *Lactuca* spp. compared to NLRs, where 70% of *RLK*s in each *Lactuca* species were homologous to another RLK from at least one sister species (Fig. 5a: right). Compared to the BUSCO completeness, the RLK ratio (1.25: 1.00: 1.00) showed an increase of *RLKs* in *L. sativa*, suggesting a possible expansion of the *RLK* family. The majority of expansions in *L. sativa* were due to *G-LecRK*, followed by *Malectin-RK* and *WAK*, while other types of *RLK*s were either similar in all species or slightly inflated in *L. sativa*. The extra *G-LecRK* and *WAK* copies might confer specific immunity in *L. sativa*. For example, G-LecRK and WAK can both mediate resistance to *Phytophthora* spp. (oomycete) in tobacco and melon plants (Wang *et al.* 2020; Pi *et al.* 2022). On the contrary, the expansion of *Malectin-RK* might benefit pathogen invasion in *L. sativa*, like the increased susceptibility to *Hyaloperonospora arabidopsidis* (oomycete) observed in *Arabidopsis* (Hok *et al.* 2011). Similar to *NLR*s, *RLK*s also commonly expand via tandem duplications. For example, a *G-LecRK* expansion was reported in soybean (Rodgers-Melnick *et al.* 2012; Liu *et al.* 2018). The number of tandem arrayed *RLK*s in *L. sativa* was 1.5 and 1.9 times that of the *RLK*s in *L. saligna* and *L. virosa*, respectively, which constitutes more than 60% of the difference between *L. sativa* and other 2 species (Fig. 5b: right; Supplementary Data 5b). Especially for *G-LecRK*, the number of tandem genes appeared to more than doubled in *L. sativa* (Supplementary Data 5b).

## Conclusions

Here, we present a near chromosome-level genome assembly for *L. virosa* (accession CGN04683) that has a high level of completeness. As a representative of the tertiary lettuce gene pool, this *L. virosa* genome assembly enables comparisons with *L. sativa* of the primary gene pool and *L. saligna* of the secondary gene pool. For gene content, *L. virosa* harbors a large number of genes absent from *L. saligna* and *L. sativa* and may thus constitute an important source of novel genes for lettuce breeding. Based on synteny, a 3-way genome comparison uncovered species-specific major inversions. These inversions should be considered as likely barriers to gene introgression in future breeding. In addition, we demonstrated that genome expansion in *L. virosa* is driven by the proliferation of LTR elements. An assembly-based comparison of *NLR* and *RLK* genes between *Lactuca* spp. found more immune system-related genes in the *L. sativa* genome than in those of the *L. virosa* and *L. saligna* genomes. These findings may contribute to future research on gene expression and regulation in *L. virosa.* Using this novel genome assembly, researchers can subsequently study the genetic variation in *L. virosa* populations to fully release its potential for lettuce breeding.

## Supplementary Material

jkad204_Supplementary_Data

## Data Availability

The genome assembly of *L. virosa*, is available under the BioProject PRJEB50301 (and available under CAKMRJ010000000.1 from ENA). All raw sequencing reads have been deposited in the ENA database under BioProject PRJEB56289. This includes the Illumina, PacBio, 10X, Bionano, and Hi-C whole-genome sequences as well as RNA sequencing data for genome annotation. Supplementary data are available at https://doi.org/10.4121/21900588. Supplemental material available at G3 online.
