# Supplementary material for: Genome assembly and analysis of Lactuca virosa: implications for lettuce breeding
Source: G3 (Bethesda). 2023 Sep 23;13(11):jkad204. doi: 10.1093/g3journal/jkad204 (PMC10627274; doi:10.1093/g3journal/jkad204)
Supplement: jkad204_Supplementary_Data [file jkad204_supplementary_data.zip › Supplementary_Data_Legends_G3-2023-404266.docx]

# Supplementary Data

Datasets are available in: <https://figshare.com/s/aa3ec2f08495f0d24dfb>

**Supplementary Data 1**. Genome assembly and scaffolding.

**A**, The input assembly scaffolds in the Hirise scaffolds using Hi-C. **B**, The input assembly scaffolds in the final scaffolds (WUR + BGI). **C**, The sequence length of scaffolds in final assembly (WUR + BGI).

**Supplementary Data 2**. Functional annotation and homology grouping of *L. virosa* transcripts.

**A**, Detailed match of functional annotation by different approaches for *L. virosa* transcripts for all isoforms. **B**. Detailed match of functional annotation by different approaches for *L. virosa* genes. **C**, Homology groups of the three *Lactuca* species calculated by PanTools. **D**, InterPro enrichment of *L. virosa* specific homologs.

**Supplementary Data 3**. Repeatome analysis of *L. virosa*, *L. sativa* and *L. saligna*.

**A**, Summary of RepeatMasker output for the three genomes. **B**, Matrix and cumulative stats of RepeatExplorer clusters. **C**, Curated annotation of RepeatExplorer clusters. **D,** Genomic proportion of curated clusters excluding organelle reads.

**Supplementary Data 4**. Identified NLR and RLK proteins in *L. virosa* and *L. saligna*.

**A**, Identification and classification of NLR (*L. virosa*). **B**, Identification and classification of NLR (*L. sativa*). **C**, Identification and classification of RLK (*L. virosa*). **D**, Identification and classification of RLK (*L. sativa*).

**Supplementary Data 5**. Overview of homology within NLRs and RLKs in *Lactuca*.

**A**, Homology groups of identified NLRs and RLKs for three *Lactuca* species. **B**, Tandem array detection of identified NLRs and RLKs for three *Lactuca* species.
