# Supplementary material for: Genome assembly and analysis of Lactuca virosa: implications for lettuce breeding
Source: G3 (Bethesda). 2023 Sep 23;13(11):jkad204. doi: 10.1093/g3journal/jkad204 (PMC10627274; doi:10.1093/g3journal/jkad204)
Supplement: jkad204_Supplementary_Data [file jkad204_supplementary_data.zip › Supplementary_Figure_Legends_G3-2023-404266.docx]

# Supplementary Figures

**Supplementary Figure 1**. Genome size estimation of *L. virosa* by GenomeScope. The 21-mers were counted by Jellyfish, and output was taken by GenomeScope to estimate the genome size of *L. virosa*. The frequency (y-axis) and sequencing depth (x-axis) of 21-mers are plotted. The genome size (3.3 Gbp) was estimated by the highest peak depth. len: Genome haploid length; uniq: genome unique length; het: heterozygosity rate; kcov: k-mer coverage; err: read error rate; dup: average rate of read duplication; k: k-mer length.

**Supplementary Figure 2**. K-mer and BUSCO completeness plots for the first *L. virosa* assembly. **A**, The initial assembly. **B**, The BGI *L. virosa* assembly. **C**, The final *L. virosa* assembly. **D**, BUSCO analysis of these three genomes. For the comparisons in **A**-**C**, all k-mers in the Illumina data were counted and subsequently colored according to their occurrence in the genome assembly. For **D**, eudicots_odb10 was used.

**Supplementary Figure 3**. Functional enrichment of genes unique to *L. virosa*. The top 10 most significant hits from an InterPro domain enrichment of all unique *L. virosa* genes according to homology grouping. For the full results, see Supplementary Data 2D.

**Supplementary Figure 4**. Link density histogram (generated by Dovetail Genomics). In this figure, the x and y axes give the mapping positions of the first and second read in the read pair, respectively, grouped into bins. The color of each square gives the number of read pairs within that bin. White vertical and black horizontal lines have been added to show the borders between scaffolds. Scaffolds less than 1 Mb are excluded. The red rectangle shows a potential breakage in the middle of Scaffold 5 (Hi-C assembly)/Lvir_scaffold7 (final assembly) for *L. virosa*.

**Supplementary Figure 5**. Genome composition of three *Lactuca* spp. Assemblies including and excluding N content (Supported by Supplementary table 2).

**Supplementary Figure 6**. LTR distribution at gene flanking regions (+/- 5 kb) in the *L. virosa* genome. LTR extracted from the cross_match summary (.out) file of RepeatMasker. Data were compared to the genome using bedmap to determine whether an LTR is genic or intergenic. In total, there are 10,474 genic and 1,690,664 intergenic LTRs. Among intergenic LTRs, 88,050 and 87,870 are located at up and down 5 kb regions, respectively. This bar plot shows the density of LTR for the up and down 5 kb intergenic regions (bin = 100 bp).

**Supplementary Figure 7**. Example cluster of repeat reads from RepeatExplorer. An illustration of cluster 10 (Supercluster 2) from two perspectives. The left graph shows the read contribution from three species. The right cluster demonstrates the characterized transposable elements (TEs) in this cluster.

**Supplementary Figure 8**. Circular tree of NLRs for *Lactuca* species generated by IQTREE. The tree was re-rooted at the midpoint between the TNL (NLR with TOLL/interleukin-1 receptor domain) and CNL (with coiled-coil domain) clade, and ultra-fast bootstrap approximation (UFBoot) support values were calculated with 1,000 repetitions. Branch color represents the three species: yellow for *L. sativa*, purple for *L. saligna*, and green for *L. virosa*.
