# Supplementary material for: Genome assembly and analysis of Lactuca virosa: implications for lettuce breeding
Source: G3 (Bethesda). 2023 Sep 23;13(11):jkad204. doi: 10.1093/g3journal/jkad204 (PMC10627274; doi:10.1093/g3journal/jkad204)
Supplement: jkad204_Supplementary_Data [file jkad204_supplementary_data.zip › Supplementary_Tables_G3-2023-404266.docx]

# Supplementary Tables

**Supplementary Table 1.** Summary statistics of DNA and RNA sequencing.

| **Type** | **Tissue** | **Technology** | **# reads/molecules** | **# bases** | **Genome coverage**** |
| --- | --- | --- | --- | --- | --- |
| DNA | Leaf | PacBio | 568,577 | 73,962,385,495 | 22.4X |
| DNA | Leaf | Illumina (raw) | 2,042,023,560 | 256,256,920,629 | 77.7X |
| DNA | Leaf | Illumina (Trimmed)* | 1,589,767,384 | 182,804,246,562 | 55.4X |
| DNA | Leaf | Hi-C | 898,000,000 | 134,700,000,000 | 40.8X |
| DNA | Leaf | Bionano | 5,199,031 | 684,445,635,000 | 207.4X |
| DNA | Leaf | 10X | 841,889,242 | 126,283,386,300 | 38.3X |
| RNA | Root | Illumina | 182,472,598 | 22,594,657,038 | NA |
| RNA | Leaf | Illumina | 178,858,548 | 22,182,587,010 | NA |
| RNA | Flower | Illumina | 154,864,274 | 19,196,608,198 | NA |

*Trimmed Illumina reads used for genome size estimation by GenomeScope.
**Genome coverage based on expected genome size of 3.3Gbp.

**Supplementary Table 2.** Overview of all statistics of *L. virosa* during the process of improving the assembly. N90 and L90 statistics are calculated for the scaffolded assembly.

| **Step** | **# seq** | **Total length** | **N90** | **L90** | **Longest** | **Shortest** | **# Ns** | **# Gaps** |
| --- | --- | --- | --- | --- | --- | --- | --- | --- |
| 00 input genome | 29 | 3305115164 | 278534532 | 9 | 442430716 | 90711 | 905096689 | 202872 |
| 01 polish | 29 | 3307727908 | 278766989 | 9 | 442776370 | 90711 | 904557320 | 116360 |
| 02 combine & purge | 54814 | 3569450220 | 76965895 | 11 | 442776370 | 1001 | 904854863 | 152711 |
| 03 filter non-nuclear | 54786 | 3569358957 | 76965895 | 11 | 442776370 | 1001 | 904854281 | 152696 |
| 04 filter contamination | 39955 | 3529432463 | 116478781 | 10 | 442776370 | 1001 | 904628957 | 146211 |
| 05 polish new | 39955 | 3534064810 | 116478781 | 10 | 442776370 | 930 | 903780369 | 128483 |
| 07 scaffold | 24767 | 3596785038 | 76965895 | 11 | 442776370 | 930 | 965886005 | 143368 |
| 08 filter RNA-seq | 6727 | 3449425426 | 116478781 | 10 | 442776370 | 1003 | 937474839 | 130693 |
| 09 filter length | 5855 | 3446681275 | 116478781 | 10 | 442776370 | 5010 | 937453665 | 130351 |

**Supplementary Table 3.** Summary of the different *L. virosa* assemblies used in this study.

| **Assembly version** | **Initial assembly** | **Wei et al. (2021) assembly** | **Final assembly** |
| --- | --- | --- | --- |
| **Genome size** | 3,305,115,164 | 2,912,862,072 | 3,446,681,275 |
| **# seq** | 29 | 3,694,810 | 5,855 |
| **N50** **scaffold** | 316,657,893 | 4,910 | 316,905,932 |
| **L50** **scaffold** | 5 | 137,357 | 5 |
| **N90** **scaffold** | 278,534,532 | 201 | 116,478,781 |
| **L90** **scaffold** | 9 | 1,468,356 | 10 |
| **Assembly complete BUSCO** | 75.20% | 92.70% | 96.20% |
| **# genes** | - | - | 39,887 |
| **# transcripts** | - | - | 42,791 |

**Supplementary Table 4**. Functional annotation summary*.

| **Database** | **Number of matched genes** | **Percent of total genes (%)** |
| --- | --- | --- |
| Interproscan | 31804 | 79.7 |
| SwissProt | 24362 | 61.1 |
| TrEMBL | 36941 | 92.6 |
| KEGG | 12853 | 32.2 |
| Combined | 37106 | 93.0 |

*Supported by Supplementary Data 2A-B.

**Supplementary Table 5.** Tentative linkage groups (LG) of *L. virosa* scaffolds.

| **Syntenic lettuce LG** | **Original ID** | **Short ID** | **Length** | **Orientation** |
| --- | --- | --- | --- | --- |
| Chr1 | Lvir_CGN04683_V4_scf6 | Scaffold6 | 313,930,088 | - |
| Chr1 | Lvir_CGN04683_V4_scf12 | Scaffold12 | 12,950,415 | - |
| Chr2 | Lvir_CGN04683_V4_scf5 | Scaffold5 | 316,905,932 | + |
| Chr3 | Lvir_CGN04683_V4_scf1 | Scaffold1 | 442,776,370 | - |
| Chr4 | Lvir_CGN04683_V4_scf2 | Scaffold2 | 416,659,169 | - |
| Chr4 | Lvir_CGN04683_V4_scf10 | Scaffold10 | 116,478,781 | - |
| Chr5 | Lvir_CGN04683_V4_scf3 | Scaffold3 | 385,159,916 | - |
| Chr5 | Lvir_CGN04683_V4_scf11 | Scaffold11 | 76,965,895 | - |
| Chr6 | Lvir_CGN04683_V4_scf9 | Scaffold9 | 278,766,989 | + |
| Chr7 | Lvir_CGN04683_V4_scf8 | Scaffold8 | 290,218,532 | + |
| Chr8 | Lvir_CGN04683_V4_scf4 | Scaffold4 | 342,977,835 | + |
| Chr9 | Lvir_CGN04683_V4_scf7 | Scaffold7 | 307,405,596 | - |

**Supplementary Table 6.** Percentage of sequence for different types of repeat elements in *Lactuca* spp. genomes (Supported by Supplementary Data 3A). For each species, both total percentage (Total) and percentage for the genome disregarding unknown bases (Excl N/X) are given.

| **Species** | ***L. sativa*** | |  | ***L. saligna*** | |  | ***L. virosa*** | |
| --- | --- | --- | --- | --- | --- | --- | --- | --- |
| **Classification** | **Total** | **Excl N/X**** |  | **Total*** | **Excl N/X**** |  | **Total*** | **Excl N/X**** |
| SINEs | 0.03 | 0.03 |  | 0.01 | 0.01 |  | 0.72 | 0.99 |
| LINEs | 1.24 | 1.34 |  | 0.32 | 0.34 |  | 0.31 | 0.42 |
| LTR elements | 56.73 | 61.39 |  | 57.28 | 61.24 |  | 47.51 | 65.26 |
| DNA elements | 3.74 | 4.04 |  | 2.97 | 3.17 |  | 2.88 | 3.96 |
| Unclassified | 12.53 | 13.56 |  | 16.42 | 17.55 |  | 8.92 | 12.25 |
| Total TEs | 74.27 | 80.37 |  | 76.99 | 82.32 |  | 60.34 | 82.88 |
|  |  |  |  |  |  |  |  |  |
| Small RNA: | 0.03 | 0.04 |  | 0.17 | 0.19 |  | 0.17 | 0.24 |
| Satellites | 0.79 | 0.86 |  | 0.86 | 0.92 |  | 1.16 | 1.59 |
| Simple repeats | 0.78 | 0.85 |  | 0.12 | 0.13 |  | 0.07 | 0.09 |
| Low complexity | 0.00 | 0.00 |  | 0 | 0 |  | 0 | 0 |

* Percentage of annotated repeat sequence calculated based on total genome length.

** Percentage of annotated repeat sequence calculated based on genome length excluding N/X content.

**Supplementary Table 7.** Summary of individual and comparative RepeatExplorer analysis. Lsat is *L. sativa*, Lsal is *L. saligna* and Lvir is *L. virosa*.

| **Analysis** | **Individual** | | | **Comparison** |
| --- | --- | --- | --- | --- |
| **Sample** | **Lsat** | **Lsal** | **Lvir** | **Lsat + Lsal + Lvir** |
| Coverage of sampled reads (x) | 0.2 | 0.2 | 0.2 | 0.07 |
| Number of input reads | 4,166,668 | 3,833,334 | 6,166,668 | 4,958,336 |
| Number of analyzed reads | 3,933,112 | 3,651,552 | 3,865,971 | 4,830,991** |
| Coverage of analyzed reads (x) | 0.19 | 0.19 | 0.13 | 0.07 |
| Number of singlets | 814,812 | 827,510 | 685,087 | 982,735 |
| Reads in top clusters (%) | 68 | 65 | 74 | 71 |
| Reads in all clusters (%) | 79 | 77 | 82 | 80 |
| # top-clusters* | 264 | 245 | 239 | 266 |
| # superclusters | 162 | 150 | 153 | 171 |

* Cluster size > 0.01% analyzed reads.

** SAL = 1,307,006, SAT = 1,420,966, and VIR = 2,103,018.

**Supplementary Table 8.** Genomic proportion of annotated clusters for individual and comparative analyses. Lsat is *L. sativa*, Lsal is *L. saligna* and Lvir is *L. virosa*.

| **Mobile element classification** | | |  | **Genomic proportion* (%)** | | | |
| --- | --- | --- | --- | --- | --- | --- | --- |
|  |  |  |  | **Lsat** | **Lsal** | **Lvir** | **Comparison** |
| Class I | LTR_Gypsy | Tekay |  | 29.71 | 25.01 | 33.64 | 33.60 |
|  |  | Athila |  | 0.83 | 1.77 | 0.79 | 1.68 |
|  |  | Retand |  | 0.06 | 0 | 0.32 | 0.19 |
|  |  | CRM |  | 0.16 | 0.06 | 0.11 | 0.12 |
|  |  | Galadriel |  | 0.01 | 0 | 0.00 | 0.00 |
|  |  | Other chromovirus |  | 0.09 | 0.07 | 0.24 | 0.08 |
|  |  | Other |  | 5.3 | 9.14 | 2.78 | 2.43 |
|  | LTR_Copia | Angela |  | 16.62 | 16.21 | 19.59 | 18.18 |
|  |  | SIRE |  | 6.66 | 4.32 | 7.50 | 6.34 |
|  |  | Ivana |  | 0 | 0 | 0.02 | 0.02 |
|  |  | Ale |  | 0.05 | 0 | 0.04 | 0.05 |
|  |  | Bianca |  | 0.05 | 0.12 | 0.08 | 0.09 |
|  |  | Tork |  | 0.03 | 0 | 0.20 | 0.11 |
|  |  | TAR |  | 0.23 | 0.22 | 0.24 | 0.23 |
|  |  | Alesia |  | 0 | 0.02 | 0.00 | 0.01 |
|  |  | Ikeros |  | 0 | 0 | 0.01 | 0.02 |
|  |  | Other |  | 1.17 | 0.4 | 1.37 | 1.07 |
|  | Other LTR |  |  | 0.27 | 1.3 | 1.41 | 0.80 |
|  | LINE |  |  | 0.42 | 0.2 | 0.04 | 0.05 |
|  | SINE |  |  | 0.02 | 0 | 0.00 | 0.00 |
|  | Pararetrovirus |  |  | 0 | 0 | 0.03 | 0.00 |
| Class II | DNA transposon | EnSpm_CACTA |  | 0.13 | 0.09 | 0.23 | 0.21 |
|  |  | hAT |  | 0.13 | 0.11 | 0.08 | 0.11 |
|  |  | MuDR_Mutator |  | 0.17 | 0.22 | 0.05 | 0.15 |
|  |  | PIF_Harbinger |  | 0.16 | 0.15 | 0.08 | 0.18 |
|  |  | MITE |  | 0.5 | 0.51 | 0.40 | 0.46 |
|  |  | Helitron |  | 0.17 | 0.21 | 0.05 | 0.15 |
|  |  | Other |  | 0.03 | 0 | 0.00 | 0.00 |
| Other | Satellite DNA |  |  | 0.06 | 0.58 | 0.69 | 0.65 |
|  | rDNA |  |  | 0.5 | 1.03 | 0.39 | 0.56 |
|  | Unknown |  |  | 3.06 | 2.18 | 2.00 | 1.91 |
| Total | annotated** |  |  | 63.54 | 61.75 | 70.40 | 67.54 |
|  | all |  |  | 66.61 | 63.93 | 72.40 | 69.45 |

 * Organelle reads (201,227 out of 4,830,991 analyzed reads) excluded before calculation based on the curated annotation from Supplementary Data 3C and genomic proportion for each type of repeat element from Supplementary Data 3D.

** Excluding unannotated repeats in “Other” group.

**Supplementary Table 9.** Genomic proportion of six groups after hierarchical clustering for annotated repeat clusters (supports Figure 4).

| **Mobile element classification** | | |  | **Genomic proportion* (%)** | | | | | |
| --- | --- | --- | --- | --- | --- | --- | --- | --- | --- |
|  |  |  |  | **Lsat_D1** | **Lsat_D2** | **Lsal_D1** | **Lsal_D2** | **Lvir_D1** | **Lvir_D2** |
| Class I | LTR_Gypsy | Tekay |  | 1.07 | 1.46 | 3.75 | 0.32 | 2.53 | 24.47 |
|  |  | Athila |  | 0.75 | 0.00 | 0.33 | 0.17 | 0.04 | 0.39 |
|  |  | Retand |  | 0.00 | 0.00 | 0.00 | 0.00 | 0.00 | 0.19 |
|  |  | CRM |  | 0.07 | 0.00 | 0.00 | 0.00 | 0.00 | 0.05 |
|  |  | chromovirus |  | 0.00 | 0.00 | 0.00 | 0.00 | 0.00 | 0.08 |
|  |  | Other |  | 0.00 | 0.05 | 0.24 | 0.00 | 0.00 | 2.13 |
|  | LTR_Copia | Angela |  | 2.23 | 0.00 | 0.00 | 0.00 | 0.39 | 15.55 |
|  |  | SIRE |  | 1.27 | 1.41 | 0.00 | 0.00 | 0.16 | 3.50 |
|  |  | Ivana |  | 0.00 | 0.00 | 0.00 | 0.00 | 0.02 | 0.00 |
|  |  | Ale |  | 0.00 | 0.00 | 0.00 | 0.00 | 0.00 | 0.05 |
|  |  | Bianca |  | 0.00 | 0.00 | 0.00 | 0.00 | 0.09 | 0.00 |
|  |  | Tork |  | 0.00 | 0.00 | 0.00 | 0.00 | 0.00 | 0.11 |
|  |  | TAR |  | 0.00 | 0.00 | 0.00 | 0.00 | 0.03 | 0.20 |
|  |  | Alesia |  | 0.00 | 0.00 | 0.00 | 0.01 | 0.00 | 0.00 |
|  |  | Ikeros |  | 0.00 | 0.02 | 0.00 | 0.00 | 0.00 | 0.00 |
|  |  | Other |  | 0.00 | 0.00 | 0.00 | 0.00 | 0.02 | 1.04 |
|  | Other LTR |  |  | 0.00 | 0.10 | 0.00 | 0.00 | 0.01 | 0.70 |
|  | LINE |  |  | 0.00 | 0.00 | 0.00 | 0.00 | 0.00 | 0.05 |
| Class II | DNA transposon | EnSpm_CACTA |  | 0.08 | 0.00 | 0.00 | 0.00 | 0.00 | 0.12 |
|  |  | hAT |  | 0.01 | 0.07 | 0.00 | 0.01 | 0.01 | 0.01 |
|  |  | MuDR_Mutator |  | 0.14 | 0.00 | 0.00 | 0.00 | 0.00 | 0.02 |
|  |  | PIF_Harbinger |  | 0.00 | 0.06 | 0.00 | 0.01 | 0.08 | 0.02 |
|  |  | MITE |  | 0.06 | 0.07 | 0.08 | 0.02 | 0.12 | 0.11 |
|  |  | Helitron |  | 0.10 | 0.00 | 0.03 | 0.02 | 0.00 | 0.00 |
| Other | SatDNA |  |  | 0.00 | 0.36 | 0.00 | 0.09 | 0.06 | 0.14 |
|  | rDNA |  |  | 0.00 | 0.00 | 0.00 | 0.56 | 0.00 | 0.00 |
|  | Unannotated |  |  | 0.32 | 0.20 | 0.13 | 0.03 | 0.12 | 1.11 |
| Total | Annotated** |  |  | 5.77 | 3.60 | 4.43 | 1.23 | 3.57 | 48.93 |
|  | all |  |  | 6.09 | 3.80 | 4.56 | 1.26 | 3.69 | 50.05 |

* Organelle reads excluded before calculation.

** Excluding unannotated repeats in “Other” group.

**Supplementary Table 10.** Summary of NLR domain search for three *Lactuca* spp. (Supports Table 2).

| **Type** | **Structure**** | **Number of gene** | | |
| --- | --- | --- | --- | --- |
|  |  | ***L. sativa*** | ***L. saligna*** | ***L. virosa*** |
| CNL type* | CNL | 72 | 72 | 74 |
|  | CN | 4 | 11 | 12 |
|  | NcL | 71 | 34 | 48 |
|  | Nc | 11 | 22 | 14 |
|  | Total | 158 | 139 | 148 |
| TNL type | TNL | 184 | 133 | 106 |
|  | TN | 1 | 4 | 0 |
|  | NtL | 42 | 38 | 54 |
|  | Nt | 0 | 9 | 1 |
|  | Total | 227 | 184 | 161 |
| Total NLR | | 385 | 323 | 309 |

* RPW8 and Rx_N type of CNL included in this study.

** Capital letters represent the domain identified by HMMER search, while lowercase letters represent the NLR classification by phylogeny based on nucleotide-binding domain alignment.

**Supplementary Table 11.** Pfam HMM motifs used for RLK classification (Supports Table 2).

| **Domain** | **Pfam** | **Description** |
| --- | --- | --- |
| Pkinase | PF00069.26 | Protein kinase domain |
| B_lectin | PF01453.26 | D-mannose binding lectin |
| EGF_CA | PF07645.17 | Calcium-binding EGF domain |
| GDPD | PF03009.19 | Glycerophosphoryl diester phosphodiesterase family |
| GUB_WAK_bind | PF13947.8 | Wall-associated receptor kinase galacturonan-binding |
| Lectin_C | PF00059.23 | Lectin C-type domain |
| Lectin_legB | PF00139.21 | Legume lectin domain |
| LRR_1 | PF00560.35 | Leucine Rich Repeat |
| LRR_2 | PF07723.15 | Leucine Rich Repeat |
| LRR_3 | PF07725.14 | Leucine Rich Repeat |
| LRR_4 | PF12799.9 | Leucine Rich Repeat |
| LRR_5 | PF13306.8 | Leucine Rich Repeat |
| LRR_6 | PF13516.8 | Leucine Rich Repeat |
| LRR_8 | PF13855.8 | Leucine Rich Repeat |
| LRR_9 | PF14580.8 | Leucine Rich Repeat |
| LTP_2 | PF14368.8 | Probable lipid transfer |
| LysM | PF01476.22 | LysM domain |
| Malectin | PF11721.10 | Malectin domain |
| Malectin_like | PF12819.9 | Malectin-like domain |
| PAN_1 | PF00024.28 | PAN domain |
| PAN_2 | PF08276.13 | PAN-like domain |
| PAN_4 | PF14295.8 | PAN domain |
| PRIMA1 | PF16101.7 | Proline-rich membrane anchor 1 |
| RCC1_2 | PF13540.8 | Regulator of chromosome condensation (RCC1) repeat |
| RVT_2 | PF07727.16 | Reverse transcriptase (RNA-dependent DNA polymerase) |
| Stress-antifung | PF01657.19 | Salt stress response/antifungal |
| SWIM | PF04434.19 | SWIM zinc finger |
| S_locus_glycop | PF00954.22 | S-locus glycoprotein domain |
| Thaumatin | PF00314.19 | Thaumatin family |
| WAK | PF08488.13 | Wall-associated kinase |
| WAK_assoc | PF14380.8 | Wall-associated receptor kinase C-terminal |

**Supplementary Table 12.** RLK classification based on extracellular domain (Supports Table 2).

| **Domain** | **Classification*** | | | | | | | | | |
| --- | --- | --- | --- | --- | --- | --- | --- | --- | --- | --- |
|  | **Rcc1-RK** | **WAK** | **G-LecRK** | **L-LecRK** | **C-LecRK** | **CRK** | **LysM-RK** | **Malectin-RK** | **LRR-RK** | **PERK** |
| RCC1_2 | x |  |  |  |  |  |  |  |  |  |
| GUB_WAK_bind |  | x |  |  |  |  |  |  |  |  |
| EGF_CA |  | x |  |  |  |  |  |  |  |  |
| WAK |  | x |  |  |  |  |  |  |  |  |
| WAK_assoc |  | x |  |  |  |  |  |  |  |  |
| B_lectin |  |  | x |  |  |  |  |  |  |  |
| PAN_2 |  |  | x |  |  |  |  |  |  |  |
| PAN_1 |  |  | x |  |  |  |  |  |  |  |
| PAN_4 |  |  | x |  |  |  |  |  |  |  |
| S_locus_glycop |  |  | x |  |  |  |  |  |  |  |
| Lectin_legB |  |  |  | x |  |  |  |  |  |  |
| Lectin_C |  |  |  |  | x |  |  |  |  |  |
| Stress-antifung |  |  |  |  |  | x |  |  |  |  |
| LysM |  |  |  |  |  |  | x |  |  |  |
| LRR_1 |  |  |  |  |  |  |  | x | x |  |
| LRR_2 |  |  |  |  |  |  |  | x | x |  |
| LRR_4 |  |  |  |  |  |  |  | x | x |  |
| LRR_5 |  |  |  |  |  |  |  | x | x |  |
| LRR_6 |  |  |  |  |  |  |  | x | x |  |
| LRR_8 |  |  |  |  |  |  |  | x | x |  |
| LRR_9 |  |  |  |  |  |  |  | x | x |  |
| LRR |  |  |  |  |  |  |  | x | x |  |
| Malectin |  |  |  |  |  |  |  | x |  |  |
| Malectin_like |  |  |  |  |  |  |  | x |  |  |
| PRIMA1 |  |  |  |  |  |  |  |  |  | x |

***** Abbreviation for classified RLKs:

RCC1-RK RCC1 repeat receptor-like kinase

WAK Wall-associated receptor-like kinase

G-LecRK G-type lectin receptor kinase

L-LecRK L-type lectin receptor kinase

C-LecRK C-type lectin receptor kinase

CRK cysteine-rich receptor-like kinases

LysM-RK Lysin motif receptor-like kinase

Malectin-RK Malectin receptor-like kinase

LRR-RK Leucine-rich repeat receptor-like kinase

PERK proline-rich extensin-like receptor kinase
