# Supplementary figures and images for: Genome assembly and analysis of Lactuca virosa: implications for lettuce breeding
Source: G3 (Bethesda). 2023 Sep 23;13(11):jkad204. doi: 10.1093/g3journal/jkad204 (PMC10627274; doi:10.1093/g3journal/jkad204)

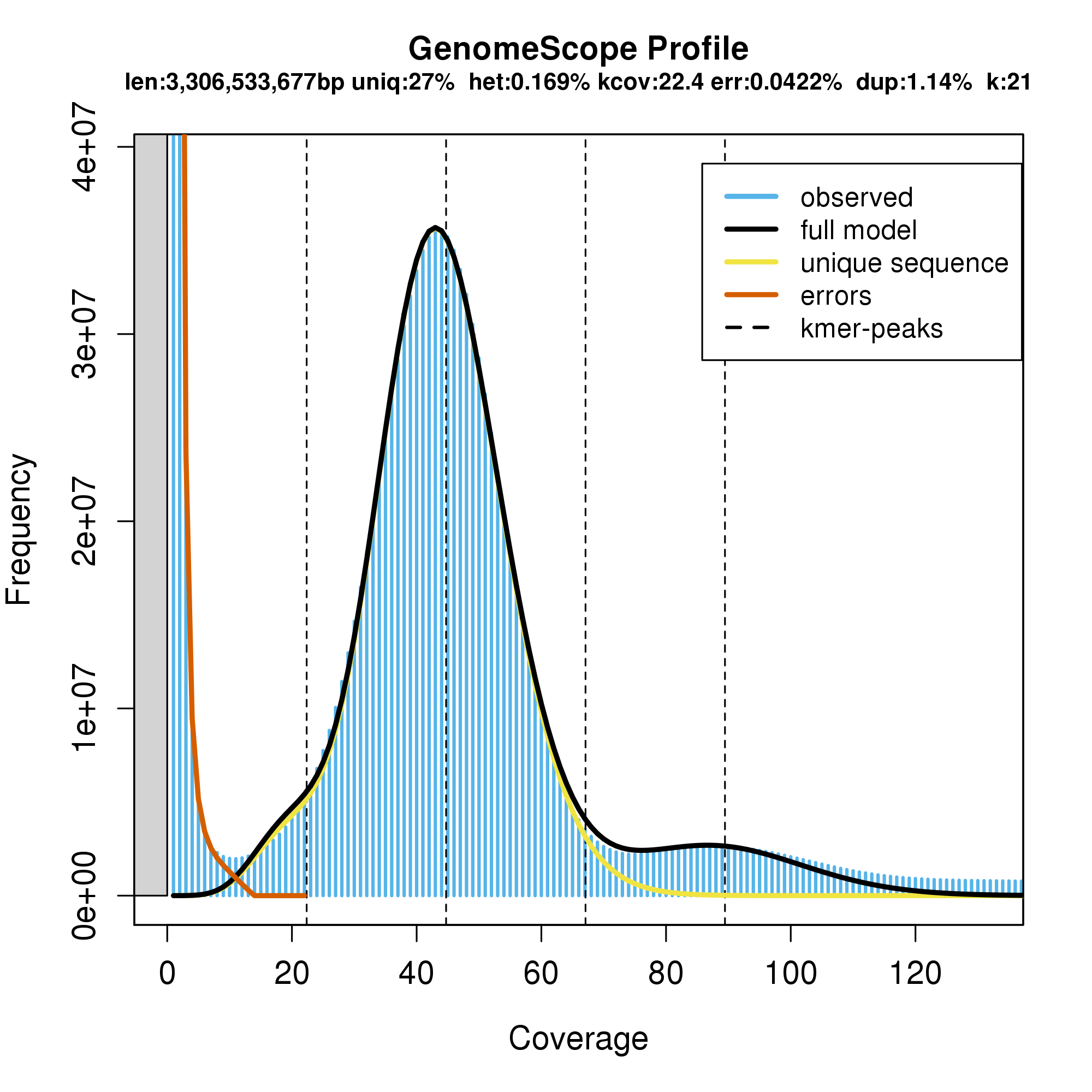

Supplement: jkad204_Supplementary_Data [file jkad204_supplementary_data.zip › Supplementary_Figure_1_G3-2023-404266.png]

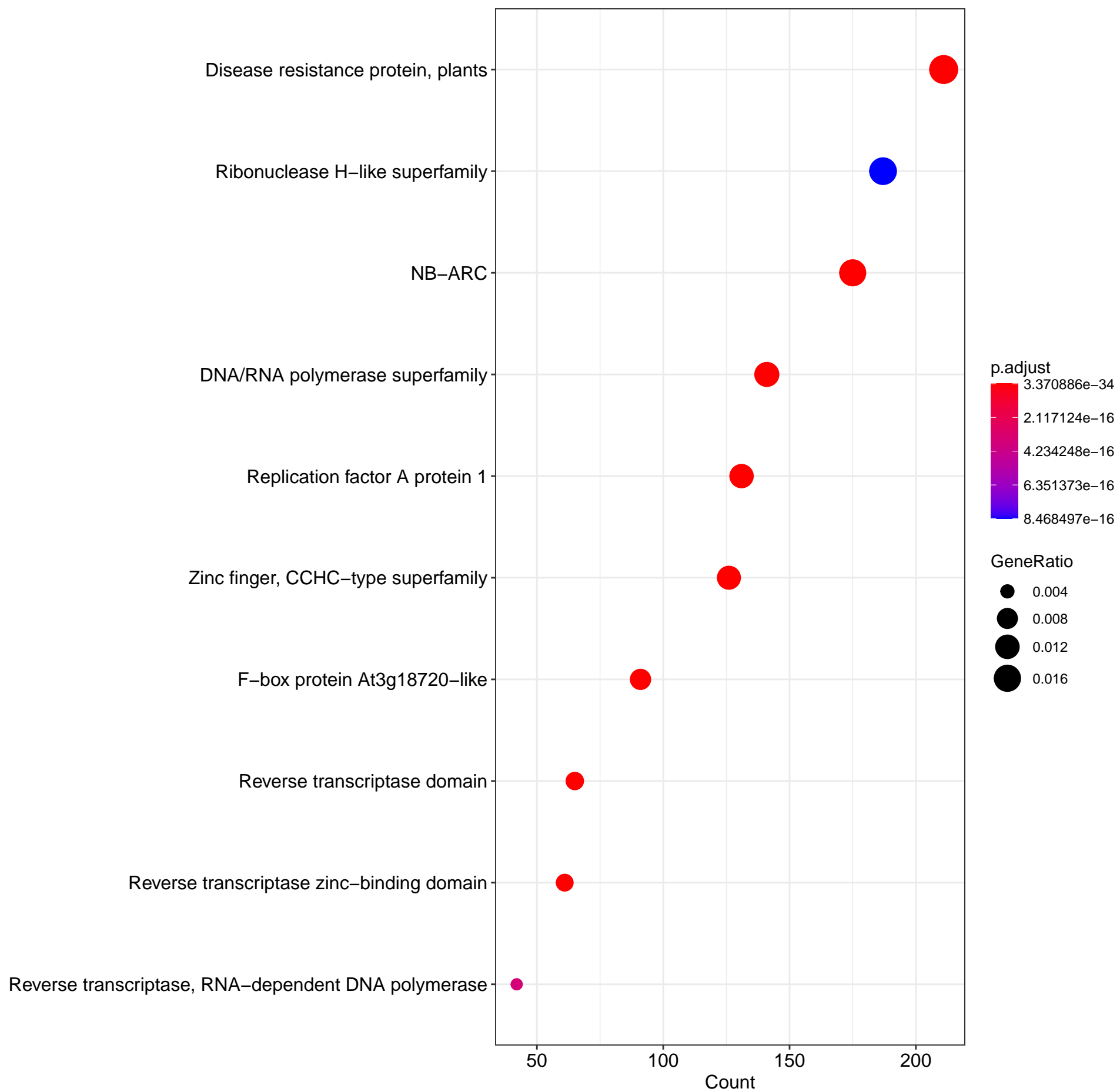

Supplement: jkad204_Supplementary_Data [file jkad204_supplementary_data.zip › Supplementary_Figure_3_G3-2023-404266.pdf]

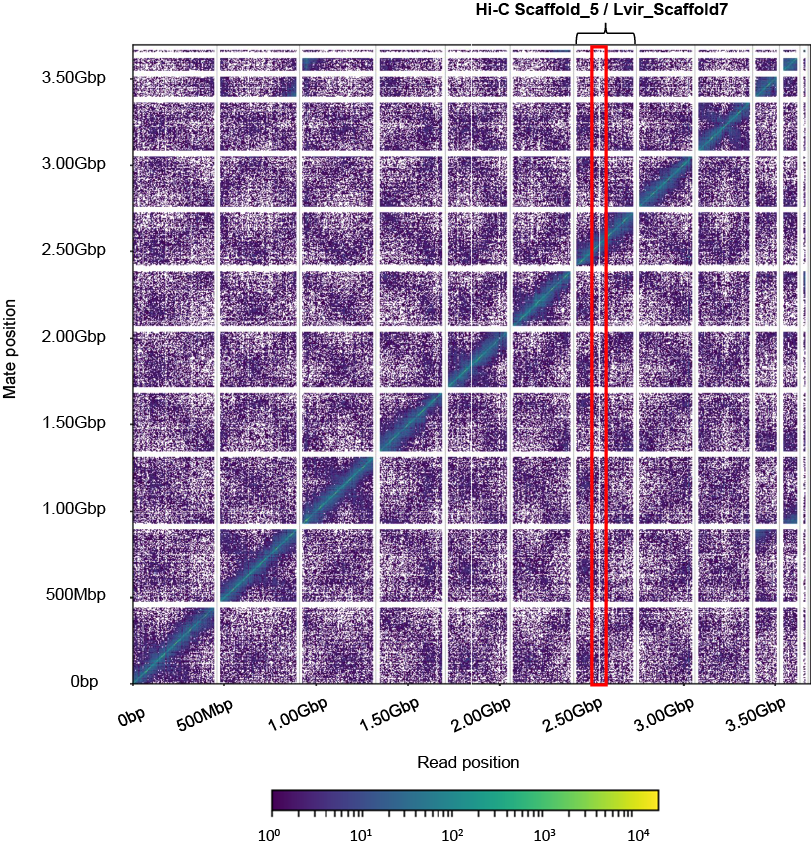

Supplement: jkad204_Supplementary_Data [file jkad204_supplementary_data.zip › Supplementary_Figure_4_G3-2023-404266.png]

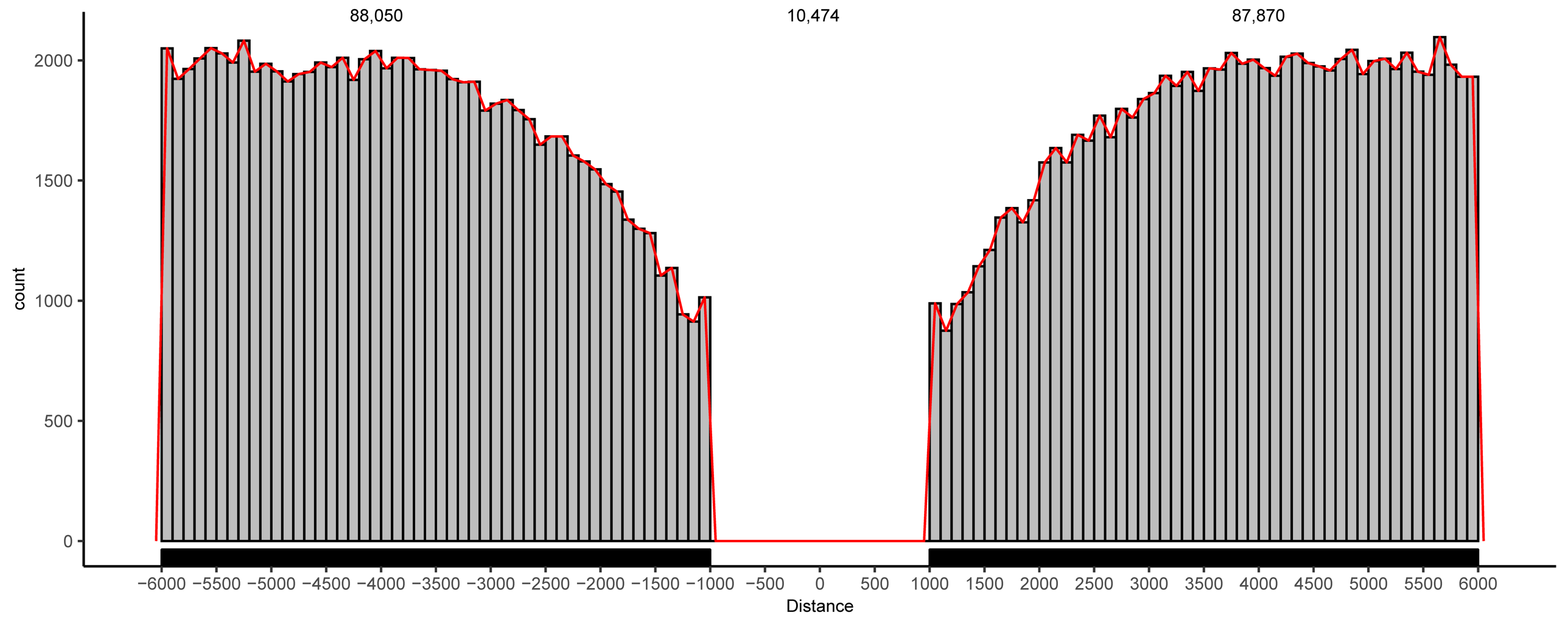

Supplement: jkad204_Supplementary_Data [file jkad204_supplementary_data.zip › Supplementary_Figure_6_G3-2023-404266.png]
